# Dexmedetomidine may benefit cognitive function after laparoscopic cholecystectomy in elderly patients

**DOI:** 10.3892/etm.2012.811

**Published:** 2012-11-13

**Authors:** JINGJUN CHEN, JUNQIANG YAN, XUEPING HAN

**Affiliations:** 1 Departments of Anesthesiology, The First Affiliated Hospital of Henan University of Science and Technology, Luoyang, Henan 471003;; 2 Neurology, The First Affiliated Hospital of Henan University of Science and Technology, Luoyang, Henan 471003;; 3 Department of Anesthesiology, The First Affiliated Hospital of Zhengzhou University, Zhengzhou, Henan 450052, P.R. China

**Keywords:** dexmedetomidine, cognitive function, laparoscopic cholecystectomy

## Abstract

Laparoscopic cholecystectomy is performed with increasing frequency in aging populations. However, in elderly patients, cognitive dysfunction following surgery may impair the outcome of surgical procedures. Dexmedetomidine (DEX) has been demonstrated to have a neuroprotectve effect in animal experiments. However, it is unclear whether DEX also has a neuroprotective effect in human patients. The present study was a randomized, placebo-controlled double-blind trial of 126 patients who had undergone laparoscopic cholecystectomy, using clinical interviews to determine whether intravenously administrated DEX during general anesthesia ameliorates cognitive function impairment. The cognitive deficit of each patient was assessed using the Mini-Mental State Examination (MMSE). The scores on the MMSE for the DEX and control groups one week after surgery (DEX group, 27.6±1.2; control group, 25.7±1.5) were significantly different (P=0.005). The MMSE scores of patients ≤65 years old were significantly higher than those of patients >65 one week after surgery. The MMSE scores were significantly different between the two age groups in the control patients (≤65 years old, 28.3±1.2; >65 years old, 26.6±2.1; P=0.036), while the difference was not statistically significant in the DEX-treated patients. Eight patients in the DEX group and 15 patients in the control group had mild cognitive impairment (26≥ MMSE score ≥21) although the difference was not statistically significant. The findings of the present study support the hypothesis that DEX administration may be an effective method for ameliorating postoperative cognitive impairment in elderly patients who have undergone laparoscopic cholecystectomy. Further research is required to confirm the findings of the present study.

## Introduction

Postoperative cognitive impairment varies from mild memory loss to an inability to concentrate or process information. Price *et al*(1) analyzed the severity of cognitive function impairment in 77 patients three months after noncardiac surgery and revealed that executive dysfunction and combined memory impairment were associated with significant functional limitations, whereas patients with only memory decline did not exhibit functional impairment. Bedford (2) retrospectively reviewed 1,193 elderly patients who underwent surgery under general anesthesia during a five-year period and observed that ∼10% of older patients had cognitive problems following surgery. The majority of these patients experienced mild problems after surgery but remained able to function independently. Cognitive function impairment is more common in elderly than in younger patients (3).

The mechanisms responsible for postoperative cognitive decline following noncardiac surgery are unknown but potential risk factors are classified into patient, surgical and anesthetic categories. The risk of cognitive function impairment has been associated with age and the type of surgery, with a low incidence in minor surgery. Bedford (2) concluded that the cognitive problems were associated with anesthetic agents. Regional anesthesia does not appear to reduce the incidence of cognitive function impairment (4) and general anesthesia (GA) itself is considered to possibly contribute significantly to cognitive function impairment (5–9). The extent of patient recovery from the residual effects of anesthetics may be determined by clinical, psychomotor and cognitive tests (10). Cognitive function impairment occurs often and consistently, particularly in the elderly (11).

Postoperative cognitive decline in elderly individuals has been reported for over a century, however, there is a lack of consensus as to whether it directly causes permanent cognitive loss. Subsequent studies conducted on patients following noncardiac surgery generally agree that postoperative cognitive decline is quite common in the short term (up to several weeks after surgery) (4,12–13).

Dexmedetomidine (DEX), a highly selective α_2_-receptor agonist, provides excellent sedation and analgesia with minimal respiratory depression (14). It may be a useful adjuvant during general anesthesia by promoting hemodynamic stability (15) and decreasing the required doses of anesthetics and analgesics (16–17). Recent multicenter trials indicate that it is an effective baseline sedative and results in greater patient satisfaction, less opioid requirement and less respiratory depression compared with a placebo (18–19). Our previous study (20) demonstrated that adrenergic receptors are important in cognition. All memory, learning and selective attention is affected by increased dorsal noradrenergic bundles from the locus ceruleus in the mesocephalon, where α_2_-adrenoceptors (α_2_-AR) are produced. In the frontal lobe, α_2_-ARs also mediate increased attention (21–22). In addition to inducing sedation and analgesia, α_2_-ARs also have central effects and alter cortical arousal (23).

The purpose of the present study was to evaluate the effects of the intravenous (IV) administration of DEX during general anesthesia on postoperative cognitive function in elderly Chinese patients who had undergone laparoscopic cholecystectomy.

## Patients and methods

### Patients

The present randomized, placebo-controlled double-blind trial was carried out at the Departments of Anesthesia and Neurology of the First Affiliated Hospital of Henan University of Science and Technology (LuoYang, China) between July 2009 and December 2011. Experienced neurologists and anesthetists performed the evaluations and completed the examination of the inpatients. The 126 patients who had undergone laparoscopic cholecystectomy with general anesthesia were randomly allocated into two groups: the DEX group (n=63) and the normal saline group (control group, n=63), prior to anesthesia induction by the sealed envelope method. The patients and anesthesiologists were blinded to whether the patient was receiving DEX or placebo.

The patient exclusion criteria were: i) ages <60 or >75 years; ii) preoperative hypotension (mean arterial blood pressure <60 mmHg); iii) preoperative bradycardia (heart rate <45 beats/min); iv) disease of the central nervous system; v) history of mental illness, recent use of sedatives or analgesics and impaired sensation; and vi) refusal to participate in the study. Cognitive function was evaluated with the Mini-Mental State Examination (MMSE) (24). All scales were available and validated for the Chinese population. The demographics and clinical data of the subjects are shown in Table I.

### Anesthetic management

Anesthetic management was standardized. Anesthesia was induced with propofol (2 mg/kg), fentanyl (3–5 μg/kg) and midazolam (0.04–0.05 mg/kg). Vecuronium (0.1 mg/kg) was used to facilitate tracheal intubation. Endotracheal intubation was achieved with an appropriately sized endotracheal tube. After the induction of general anesthesia, the DEX group patients received an initial DEX dose of 1 mg/kg over 10 min, followed by a continuous infusion at 0.4 mg/kg/h until the end of surgery. The control group received a placebo infusion of normal saline. General anesthesia was maintained with remifentanil (10–12 μg/kg/h) and propofol (4–6 μg/kg/h). In the preoperation room, all the vital signs were observed and recorded by computer by a staff nurse. In the operating theater, all the baseline parameters, including heart rate (HR), electrocardiography (ECG), mean arterial blood pressure (MAP), pulse oximetry (SpO_2_) and end tidal carbon dioxide (EtCO_2_), were continuously monitored throughout the surgery (Table II) and IV access was secured with an 18G cannula. A decrease in MAP of >20% below the preanesthetic baseline level was corrected by ephedrine.

Propofol was discontinued for five min prior to the end of surgery. At the end of surgery, the infusion of DEX or placebo was stopped, remifentanil was discontinued and the patient’s lungs were ventilated with 100% oxygen. Neuromuscular blockade was reversed with an IV administration of neostig-mine (0.04 mg/kg) and atropine (0.02 mg/kg) when the patients opened their eyes. Once the patients achieved a regular breathing pattern and were able to follow a verbal command to squeeze the anesthesiologist’s hand, tracheal extubation was performed.

### Ethics statement

The local ethics committee of the First Affiliated Hospital of Henan University of Science and Technology approved the study which was conducted according to the principles expressed in the Declaration of Helsinki. The patients were informed that DEX or a placebo would be used in anesthesia and they gave written informed consent prior to the operation. This consent was verbal for the measurements of MMSE in the present study.

### Statistical analysis

All the data for continuous variables (age, disease duration, HR, MAP, SpO_2_ and MMSE) are shown as the mean ± standard deviation and the categorical variable (gender) is shown as a percentage. The total scores on the MMSE were calculated as the sum of single items. A student’s t-test was used to compare the differences in the heart rate, mean arterial blood pressure and MMSE scores between pre- and post-surgery. The Chi-square test was used to test for statistical differences in the ratio of cognitive impairment between the two groups. The SPSS 13.0 software (IBM, Chicago, IL, USA) was used for the statistical analyses. P<0.05 was considered to indicate a statistically significant difference.

## Results

### Patients and clinical parameters

The two groups were comparable with regard to the distributions of age, weight, height, gender, American Society of Anesthesiologists (ASA) grade, duration of anesthesia and duration of surgery and exhibited non-significant differences upon statistical comparison. The surgical procedures performed in the present study were compatible with each other. The HR, MAP, SpO_2_ and EtCO_2_ before the interruption of the maintenance of general anesthetics were not significantly different. The total doses of intraoperative remifentanil, vecuronium and propofol were similar in the two groups.

In total, 122 subjects (four patients of the DEX group dropped out), including 59 patients [33 (55.9%) males and 26 (44.1%) females] who received DEX in general anesthesia and 63 patients [31 (49.2%) males and 32 (50.8%) females] in the control group (Table I), were enrolled in the present study. The mean ages of the DEX group and the control group patients were 66.2±7.5 years (range, 61–73) and 67.9±6.6 years (range, 60–72), respectively. The mean disease duration was 2.2±1.7 years (range, 0.08–4.6) for the DEX group and 2.3±1.5 years (range, 0.06–4.3) for the control group. The mean duration of surgery was 1.5±0.5 h (range, 0.9–2.1) for the DEX group and 1.4±0.6 h (range, 0.7–2.2) for the control group. The details of the demographics of all 122 patients are shown in Table I.

### Comparison of HR, MAP and SpO_2_

The preoperative mean HR and MAP were comparable between the two groups and were not statistically significantly different. The HR and MAP after surgery were observed to be significantly different by statistical analysis (Table II). However, the HR and MAP were significantly different (P= 0.009 and P= 0.015) between the DEX and control groups in the postoperative stage, with the DEX group patients having a lower mean HR and MAP than the control group patients. MAP fluctuations were minimal in the DEX group compared with those in the control group during the period ranging from extubation to recovery in the postanesthesia care unit. The other vital parameters, such as respiratory rate, SpO_2_, EtCO_2_, general consciousness level and alertness, were similar. The systolic blood pressure increased to ∼175 mmHg in three patients of the control group during the initial phase but the effect was transient and required no treatment. Severe hypotension (MAP <60 mmHg) or bradycardia (HR <40 bpm) were not observed in any subjects.

### Comparison of MMSE scores

Table III shows the preoperative and postoperative cognitive function evaluated using the MMSE. The MMSE scores before surgery (DEX group, 28.2±0.8; control group, 28.5±1.1) and one month after surgery (DEX group, 28.1±1.1; control group, 28.3±1.2) were not affected by the IV administration of DEX. However, the MMSE scores one week after surgery (DEX group, 27.6±1.2; control group, 25.7±1.5) were significantly different (P=0.005) between the two groups.

When all the subjects were divided into two groups (age <60, age >60), the MMSE scores before surgery and one month after surgery were not significantly different in the DEX group. However, the MMSE scores one week after surgery were significantly higher for patients ≤65 years old than for those >65 (Table IV). The MMSE scores were significantly higher for patients ≤65 years old than for those >65 (28.3±1.2 vs. 26.6±2.1, P=0.036, Table IV) in the control group. The difference was not statistically significant in the DEX group.

Eight patients in the DEX group and 15 patients in the control group had mild cognitive impairment (26≥MMSE score≥21). One patient in the DEX group and two patients in the control group had moderate cognitive impairment. However, the difference was not statistically significant (Table V). None of the patients had severe cognitive impairment.

## Discussion

A number of studies have investigated cognitive function following surgery, predominantly in the elderly (25–27). Cognitive function has received significant attention in terms of diagnosis, therapy and evaluation. The primary goals of the present study were to compare elderly Chinese patients who had undergone laparoscopic cholecystectomy by systematically collecting information on demographics, including age and disease duration, side effects and MMSE scores before and after surgery for the two groups. To the best of the authors’ knowledge, the present study is the first to evaluate the effect of DEX on cognitive function in elderly Chinese patients who have undergone laparoscopic cholecystectomy, using unified and integrated scales (MMSE).

In the present study, the DEX group exhibited a number of differences from the control group. The HR and MAP were not observed to be significantly different before GA in the Chinese patients who had undergone laparoscopic cholecystectomy. Notably, as shown in Table II, the HR and MAP in the control group were significantly higher than in the DEX group following the interruption of GA. This observation has also been documented in other studies (15, 28–32). DEX produced a predictable hemodynamic decline (decreased arterial blood pressure and heart rate) following surgery (33–38). There was no clinically apparent respiratory depression after the cessation of assisted ventilation. Kang *et al*(39) observed that the changes in MAP during surgery were significantly lower in DEX-treated patients than in the control, which suggests that intraoperative IV administration of DEX produces more stable hemodynamics than are observed in control patients.

The MMSE scores before surgery and one month after surgery were not significantly different. Notably, a significant reduction in the MMSE scores was observed in the control group compared with the DEX group (DEX group, mean = 27.6; control group, mean = 25.7; P=0.005; Table III) one week after surgery. This result demonstrated that, compared with the DEX group, the control group patients had already undergone a decline in cognitive function by the time they visited a neurologist one week after surgery which may imply that DEX has a neuroprotective effect. Sato *et al*(40) demonstrated in animal experiments that DEX provides neuroprotective effects by attenuating neuronal damage in the hippocampal CA1 region. In an *in vivo* experiment (41), DEX dose-dependently prevented isoflurane-induced injury in the hippocampus, thalamus and cortex in the developing brain, providing neurocognitive protection. This has also been reported in several other previous studies (42–44). If isoflurane-induced neuroapoptosis proves to be a clinical problem, the administration of DEX may be an important adjunct to prevent isoflurane-induced neurotoxicity.

Notably, when the subjects were separated into two groups according to age (≤65 and >65 years), the MMSE scores before and one month after surgery were not significantly different between the two age groups in the DEX-treated and control groups, whereas in the control group, the MMSE scores one week after surgery were significantly lower for patients >65 years old than for those ≤65 (P=0.005; Table IV). Although the reasons leading to the decline in MMSE scores are unclear, hemodynamic changes may be significant. Hemodynamic decline during the surgery causes hypoxic-ischemic brain damage which is more serious in patients >65 years than in those under. Hypoxic-ischemic brain damage may directly lead to a decline in MMSE scores but DEX causes a moderate increase in blood pressure and heart rate, decreasing hypoxic-ischemic brain damage. One month after surgery, the MMSE scores of patients of different ages were not significantly different since the hypoxic-ischemic brain damage caused by GA had been resolved. In addition, the cause of postoperative cognitive dysfunction (POCD) in elderly patients is likely to be multi-factorial and may relate to the preoperative health status of the patient, perioperative events associated with the surgery itself and, possibly, neurotoxic effects of anesthetic agents. Although the mechanism of the neuroprotection by the α_2_-agonist has not been fully elucidated, putative mechanisms include an increase in epinephrine secretion, an increase in the expression of focal adhesion kinase and the modulation of central glutamate release. In addition, a decrease in the sympathetic tone with DEX may be protective by its downregulation of inflammatory neurodegenerative processes.

The first large prospective study (45) revealed that POCD occurred in 25% of the patients at hospital discharge and 10% had measurable cognitive changes at three months after major surgery. Monk *et al*(3) evaluated adults of all ages who had undergone major noncardiac surgery and observed that only the elderly (>60 years old) were at significant risk (13%) of POCD at three months after surgery. Moller *et al*(45) identified a significant correlation between POCD one week after surgery and age by multiple logistic regression analysis; the incidence of POCD was 25.8% (95% CI, 23.1–28.5) one week after surgery. Rasmussen *et al*(46) studied 35 patients aged 60 years or older who had undergone abdominal surgery with general anesthesia, with neuropsychological tests performed before surgery and at discharge. The authors also demonstrated that age was a risk factor for postoperative cognitive function. In the present study, 15–25% of elderly Chinese patients had mild to moderate cognitive impairment following laparoscopic cholecystectomy which was consistent with the results of Moller *et al*(45). Notably, in the present study the cognitive function was perfectly normal one month after surgery, while the incidence of POCD was 9.9% at three months after surgery in the Moller *et al* study which may be associated with the type of surgery. Since laparoscopic cholecystectomy is a minor surgery that may cause mild cognitive function impairment, the cognitive impairment was completely resolved.

No patients required intervention for bradycardia or hypotension. The patients were administered atropine (1 mg) as a premedication prior to surgery to prevent DEX- induced bradycardia. With the exception of a few patients who experienced treatable nausea and vomiting, no other severe adverse effects were observed. These findings support the safety of the IV administration of DEX.

The present study had several limitations: i) only a small number of patients (DEX group, n=59; control group, n=63) were recruited and the disease duration was relatively short (DEX group, 2.2 years; control group, 2.3 years); ii) to validate and complete the questionnaire, only elderly patients with a high mean MMSE score (DEX group, 28.2; control group, 28.5) prior to surgery were selected which significantly narrowed the population of the study; and iii) the postoperative administration of other drugs was not standardized which may have contributed to cognitive impairment. The population selected in the present study according to considerations i to iii may have produced a bias in the MMSE scores following surgery in the DEX and control group patients. Therefore, larger studies should be performed, expanding the present study to a broader population. In addition, the administration of drugs should be taken into account to compensate for the shortcomings of the present study.

In conclusion, the findings of the present study support the hypothesis that DEX increases the MMSE scores one week after surgery for patients who have undergone laparoscopic cholecystectomy. To the authors’ best knowledge, the present study is the first to assess cognitive function using the MMSE scale in patients who have undergone laparoscopic cholecystectomy. Cognitive function recovery may be predicted by a systemic change in MMSE scores. Although these results provide validation, caution should be taken in translating the research findings into clinical application. The decrease in MMSE scores may be associated with multiple factors. Whether or not DEX medication may be used in the management of laparoscopic cholecystectomy is thus a potential subject of future studies.

## Figures and Tables

**Table I.** Demographic parameters.

	DEX group			Control group		
Clinical parameters	Mean (SD)	Min	Max	Mean (SD)	Min	Max
Gender, n (%)						
Male	33 (55.9)	--	31 (49.2)	-	-	
Female	26 (44.1)	--	32 (50.8)	-	-	
Age (years)	66.2 (7.5)	61	73	67.9 (6.6)	60	72
Body weight (kg)	58.6 (7.9)	47	72	57.8 (8.3)	52	73
Disease duration (years)	2.2 (1.7)	0.08	4.6	2.3 (1.5)	0.06	4.3
Operation duration (h)	1.5 (0.5)	0.9	2.1	1.4 (0.6)	0.7	2.2
BMI	22.8 (2.1)	19.2	25.3	21.6 (2.7)	18.3	24.3
ASA grade (I/II), n	28/31	-	-	29/34	-	-

DEX, dexmedetomidine; BMI, body mass index; ASA, American Society of Anesthesiologists.

**Table II.** Comparison of HR, MAP and SpO_2_.

	Before GA			After the interruption of GA		
Variable	DEX group	Control group	P-value	DEX group	Control group	P-value
HR	74.3±4.2	73.1±4.5	0.617	66.4±6.6^b^	78.6±8.3	0.009
MAP	95.3±6.5	94.2±7.6	0.372	76.4±5.3^a^	85.3±8.5	0.015
SpO_2_	98.4±0.5	98.5±0.5	0.386	98.8±0.6	98.9±0.9	0.773

GA, general anaesthesia; DEX, dexmedetomidine; HR, mean heart rate; MAP, mean arterial blood pressure; SpO_2_, mean pulse oximetry.

a P<0.05 vs. control group,

b P<0.01 vs. control group.

**Table III.** Comparison of MMSE scores.

Time of test	DEX group	Control group	P-value
Before surgery	28.2±0.8	28.5±1.1	0.487
One week after surgery	27.6±1.2^a^	25.7±1.5	0.005
One month after surgery	28.1±1.1	28.3±1.2	0.556

DEX, dexmedetomidine; MMSE, Mini Mental-State Examination.

a P<0.01 vs. control group.

**Table IV.** Comparison of MMSE scores by age (years).

	DEX group			Control group		
Time of test	Age ≤65	Age >65	P-value	Age ≤65	Age >65	P-value
Before surgery	28.3±0.9	28.1±1.3	0.596	28.4±1.1	28.1±1.4	0.836
One week after surgery	27.9±1.1	27.1±1.2	0.195	28.3±1.2	26.6±2.1	0.036^a^
One month after surgery	28.2±1.2	27.9±1.3	0.600	28.5±1.1	28.2±0.9	0.673

DEX, dexmedetomidine; MMSE, Mini Mental-State Examination.

a P<0.05 vs. control group.

**Table V.** Comparison of cognitive impairment degree at 24 h after surgery.

Cognitive impairment degree	DEX group, n (%)	Control group, n (%)	P-value
Mild cognitive impairment	8 (13.6)	15 (23.8)	0.148
Moderate cognitive impairment	1 (1.7)	2 (3.2)	0.598
Severe cognitive impairment	0 (0.0)	0 (0.0)	-

DEX, dexmedetomidine.
